# Reaction Pathways and the Underlying Mechanism of Ni_4_Cu Alloy Clusters Anchored on Graphene for CO_2_ Electroreduction to Formic Acid

**DOI:** 10.3390/nano16070434

**Published:** 2026-04-01

**Authors:** Lisu Zhang, Yanbo Zou, Xingguo Wang, Qingyang Li

**Affiliations:** Xinjiang Key Laboratory for Luminescence Minerals and Optical Functional Materials, School of Physics and Electronic Engineering, Xinjiang Normal University, Urumqi 830054, China

**Keywords:** electrochemical CO_2_ reduction reaction, Ni-based clusters, alloy catalyst, density functional theory, reaction mechanism

## Abstract

The electrochemical CO_2_ reduction reaction (CO_2_RR) offers a sustainable route for converting greenhouse gases into high-value fuels; however, its efficiency has long been constrained by the thermodynamic stability of CO_2_ molecules and the competing hydrogen evolution reaction. Using density functional theory (DFT) calculations, this work systematically investigates the catalytic performance of Ni_5_ and alloy Ni_4_Cu clusters anchored on divacancy graphene (DVG) for CO_2_RR. The results demonstrate that the introduction of Cu atoms significantly enhances the interfacial binding energy between the cluster and the support (shifting from −6.2 eV to −7.5 eV). Charge density difference analysis combined with Bader charge analysis further reveals that interfacial charge transfer and the formation of Ni–C bonds serve as the electronic origin of this improved stability. Free energy calculations show that, compared to Ni_5_/DVG, Ni_4_Cu/DVG substantially reduces the energy barrier of the rate-determining step for formic acid (HCOOH) formation from 1.18 eV to 0.26 eV, thereby significantly optimizing the reaction kinetics. Crystal orbital Hamilton population (COHP) analysis demonstrates that Cu doping modulates metal–oxygen bond strength in the key *OCHO intermediate (ICOHP: Ni-O bonds at −0.697 eV/−0.976 eV vs. Cu-O bonds at −0.408 eV/−0.492 eV), optimizing the adsorption–desorption balance and steering selectivity toward HCOOH. This work elucidates the atomic-scale electronic and bonding mechanisms underlying Ni–Cu synergistic effects, providing theoretical guidance for designing efficient non-noble metal CO_2_RR electrocatalysts.

## 1. Introduction

The relentless rise in atmospheric carbon dioxide (CO_2_) concentrations, largely driven by fossil fuel combustion and large-scale deforestation, has become one of the most pressing challenges of the modern era, with profound implications for the global climate system [1]. Driven by renewable electricity, the CO_2_RR converts CO_2_ into value-added fuels and chemical feedstocks [2,3]. This strategy offers a promising pathway for mitigating greenhouse gas emissions while promoting a carbon-neutral cycle through effective CO_2_ utilization [4,5].

Among the various products of CO_2_ electroreduction, HCOOH has garnered particular attention due to its unique dual role as both a valuable chemical commodity and a promising liquid organic hydrogen carrier. As a liquid under ambient conditions, it offers significant logistical advantages over gaseous products while also serving as a commodity chemical with established applications in animal feed preservation, leather tanning, and chemical synthesis. More importantly, formic acid is increasingly recognized as a promising liquid organic hydrogen carrier. Its high volumetric hydrogen density enables safe and efficient H_2_ storage along with on-demand release via catalytic dehydrogenation. Consequently, the selective electroreduction of CO_2_ to formic acid represents a strategic pathway that bridges sustainable chemical manufacturing and the development of a hydrogen economy. Realizing this potential, however, requires precise control over the reaction pathway, which is fundamentally governed by the activation mechanism of CO_2_ on the catalyst surface.

In the CO_2_RR, the activation of CO_2_ typically necessitates the transformation of its linear geometry into a bent configuration, a critical transition that substantially reduces the activation energy barrier [6]. It is well established that CO_2_ activation on transition metal surfaces is structure-sensitive, with varying surface geometries exerting distinct influences on reactivity [7]. Over the past decades, the catalytic activity of various materials, including transition metals such as Co, Ni, Cu, Pt, Pd, Fe and metal oxides like CeO_2_, toward CO_2_ conversion has been systematically investigated [8,9,10]. These studies consistently demonstrate that CO_2_ activation is promoted by elongation of the C-O bond and increased bending of the O-C-O angle, processes driven by significant electron transfer from the metal surface to the CO_2_ molecule [11]. Among these metals, nickel (Ni) stands out as one of the most effective catalysts owing to its ability to stabilize the bent CO_2_ intermediate, thereby substantially lowering the reaction barrier [12]. Consequently, Ni-based catalysts have been widely adopted as model systems for elucidating the fundamental mechanisms underlying CO_2_ activation and reduction.

Cluster catalysts have attracted considerable attention in heterogeneous catalysis due to their uniform active sites, high atom utilization efficiency, and the synergistic interactions among adjacent metal atoms, which collectively facilitate reactant adsorption and activation [13]. For example, Liu et al. reported that Cu_4_ clusters supported on Al_2_O_3_ thin films exhibit high catalytic activity for CO_2_ conversion to CH_3_OH under mild conditions [14]. Similarly, Guo et al. demonstrated through density functional theory calculations that Cuₙ/GR clusters stabilize key reaction intermediates via multisite cooperation, enabling highly selective CO_2_ electroreduction to CH_4_ with an overpotential as low as −0.31 eV [15]. These findings collectively underscore the pivotal role of atomic-scale synergy and maximized atom utilization in governing the catalytic performance of cluster-based catalysts.

Nickel clusters, in particular, have garnered significant research interest owing to their low cost, natural abundance, and extensive application in heterogeneous catalysis, especially in CO_2_ hydrogenation and reforming reactions [16]. To prevent cluster aggregation into larger nanoparticles, the selection of an appropriate support material is of paramount importance [17]. Defective graphene, particularly with divacancy defects, not only provides robust anchoring sites that effectively suppress metal cluster migration and coalescence but also actively participates in charge transfer processes, thereby synergistically modulating the electronic structure of the supported clusters [18,19].

Alloying strategies have garnered considerable research interest in recent years owing to their efficacy in modulating electronic structures, enriching active sites, and optimizing product selectivity [13]. Introducing a secondary metal induces electronic redistribution, which alters both the geometric and electronic properties of the catalyst and fine-tunes the adsorption behavior of key intermediates such as *COOH and *CO. Li et al. constructed atomically dispersed Ni-Fe bimetallic sites on nitrogen-doped carbon and demonstrated that, compared to monometallic Ni or Fe sites, these bimetallic configurations exhibit enhanced *COOH adsorption and *CO desorption, leading to markedly improved CO selectivity and catalytic activity [20]. This finding highlights the crucial role of alloying in steering reaction pathways.

Inspired by the above research, we first establish fundamental reaction pathway models using structurally simple pure nickel clusters. Subsequently, we introduce a second metal (Cu) to construct Ni-Cu alloy clusters. Among various alloying elements, Cu elements emerge as a particularly promising candidate for alloying with Ni, owing to its unique electronic properties and well-established role in CO_2_ electroreduction. In this work, we present a density functional theory (DFT) study to systematically investigate the electrocatalytic performance of Ni_5_ and Ni_4_Cu alloy clusters supported on defective graphene for CO_2_ reduction. As a purely theoretical investigation based on first-principles calculations, this work aims to elucidate the regulatory mechanisms through which alloying modulates the adsorption behavior of key intermediates and controls reaction pathway selectivity.

## 2. Computational Methods

The first-principles calculations were performed within the framework of density functional theory (DFT) [21] using the Vienna Ab initio Simulation Package (VASP) [22,23]. The projector-augmented wave (PAW) method [24,25] was employed to describe the interaction between core ions and valence electrons, while the exchange–correlation interactions were treated using the generalized gradient approximation (GGA) in the form of the Perdew–Burke–Ernzerhof (PBE) functional [26,27]. Van der Waals interactions were accounted for using the DFT-D3 correction method of Grimme to properly describe the non-covalent interactions between the metal clusters and the graphene support. A plane-wave basis set with a kinetic energy cutoff of 400 eV was adopted for all calculations. For all graphene-supported systems, a 6 × 6 × 1 supercell of pristine graphene was constructed, into which Ni-based clusters were subsequently introduced. The supercell dimensions are 21.31 Å in length and 12.34 Å in width, which ensures sufficient separation between periodic images of the clusters to avoid spurious inter-cluster interactions that could influence the catalytic reaction. A vacuum layer of 15 Å was placed along the z-direction to eliminate artificial periodic interactions between adjacent slabs. Brillouin zone integration was performed using a Γ-centered 3 × 3 × 1 Monkhorst–Pack k-point mesh. All atomic positions were fully relaxed until the maximum residual force on each atom was less than 0.02 eV·Å^−1^, and the energy convergence criterion for electronic self-consistent field (SCF) iterations was set to 10^−5^ eV.

In this calculation, the formula for calculating the binding energy (*E*_bind_) of the catalyst model is:

*E*_bind_ = *E*_(A+B)_ − *E*_A_ − *E*_B_ (1)

where *E*_bind_ is the binding energy between A and B, *E*_(A+B)_ is the energy of A and B in a combined state, and *E*_A_ and *E*_B_ are the energies of A and B in an isolated state

The Gibbs free energy difference (Δ*G*) can be calculated as

Δ*G* = Δ*E*_elec_ + Δ*E*_zpe_ − *T*Δ*S* (2)

where Δ*E*_elec_ is the reaction energy from the DFT total energies. In addition, we calculated zero-point energy correction Δ*E*_zpe_ and entropy energy correction *T*Δ*S* of adsorbates according to the quantum mechanical harmonic approximation at 298.15 K.

All free energies of proton-coupled electron transfer (PCET) steps were calculated using the computational hydrogen electrode (CHE) model proposed by Nørskov et al. [28]. In this model, the chemical potential of a proton–electron pair (H^+^ + e^−^) at 0 V vs. RHE is set equal to half the chemical potential of a gas-phase H_2_ molecule under standard conditions, as follows:

(3) $$\frac{1}{2}\mu_{H_{2}}=\mu_{H^{+}}+\mu_{e^{-}}$$

Unless otherwise stated, all energy barriers are calculated at 0 V vs. RHE.

## 3. Results and Discussion

### 3.1. Structural Stability

**Figure 1:**
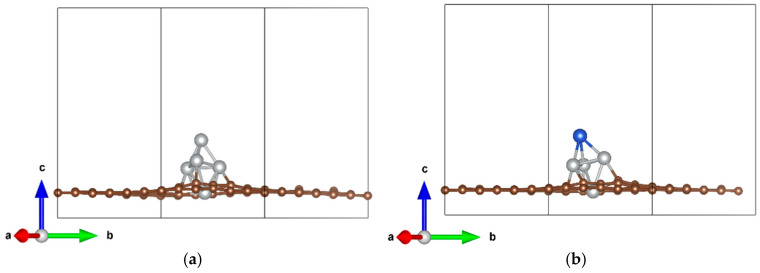
Stable configurations of the catalysts: (**a**) Ni_5_/DVG; (**b**) Ni_4_Cu/DVG. (Ni: silver, Cu: blue, C: brown).

**Figure 2:**
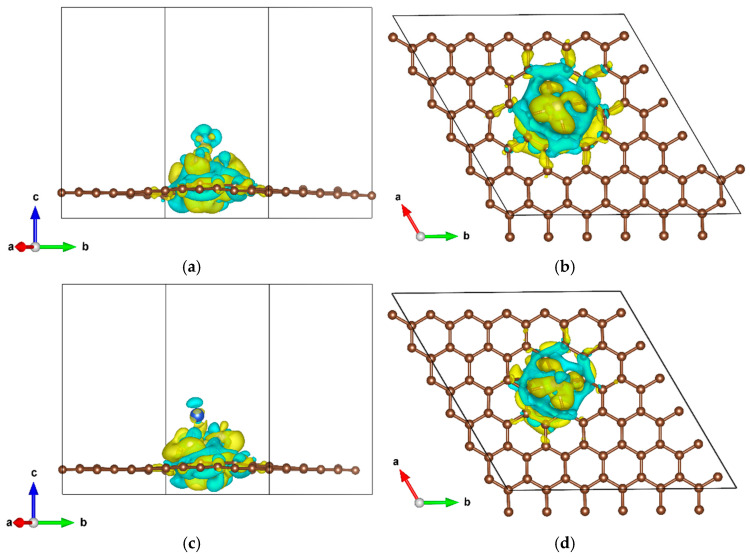
Charge density difference of the catalysts: (**a**) side view of Ni_5_/DVG; (**b**) top view of Ni_5_/DVG; (**c**) side view of Ni_4_Cu/DVG; (**d**) top view of Ni_4_Cu/DVG. The yellow and cyan isosurfaces represent charge accumulation and depletion, respectively, with an isosurface value of 0.003 e/Å^3^.

Defect sites on graphene surfaces can serve as efficient anchoring centers for metal clusters. Leveraging this principle, stable Ni_5_/DVG and Ni_4_Cu/DVG composite structures were successfully constructed in this study by precisely loading optimized Ni_5_ and Ni_4_Cu clusters onto double-vacancy defect sites (Figure 1). Calculated binding energies of −6.2 eV and −7.5 eV for Ni_5_/DVG and Ni_4_Cu/DVG, respectively, indicate that both systems form strong interfacial bonds with the substrate. Notably, Ni_4_Cu/DVG exhibits substantially enhanced thermodynamic stability, with a binding energy decrease of approximately 1.3 eV (i.e., more negative), providing a critical foundation for the design of highly stable catalysts. To further elucidate the microscopic origin of this enhanced stability, differential charge density analysis (Figure 2), the yellow and cyan regions represent charge accumulation and depletion, respectively, and Bader charge analysis were performed.

Quantitative Bader charge analysis (see Table 1) confirms net electron transfer from the metal clusters to the graphene substrate, indicating substantial charge transfer between Ni atoms and the defective carbon atoms, which gives rise to strong Ni-C interfacial interactions. This interfacial charge rearrangement and concomitant chemical bonding not only strengthen the adhesion between the support and clusters—thereby effectively suppressing surface migration and agglomeration—but also further lower the total energy of the system through charge localization and stabilization. These electronic structure features collectively account for the markedly improved structural stability observed in Ni_4_Cu/DVG.

### 3.2. Reactivity and Catalytic Performance Analysis

#### 3.2.1. Electronic Structure Analysis of CO_2_ Adsorption

**Figure 3:**
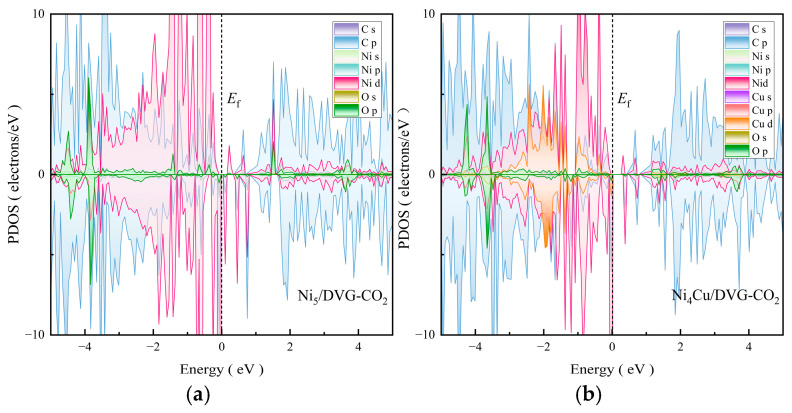
Partial density of states (PDOS) for CO_2_ adsorption. (**a**) Ni_5_/DVG (**b**) Ni_4_Cu/DVG.

Building upon the understanding of catalyst stability, we further investigated their catalytic activity, beginning with an examination of CO_2_ adsorption behavior on the Ni_5_/DVG and Ni_4_Cu/DVG surfaces. By calculating the projected density of states (PDOS) for the adsorption systems (Figure 3), we elucidated the nature of the adsorption from an electronic structure perspective. The analysis reveals that in both systems, the molecular orbitals of CO_2_—particularly the C *p* and O *p* orbitals—hybridize significantly with the metal orbitals of the catalytic active sites (Ni *d* orbitals, and Cu *d* orbitals in Ni_4_Cu/DVG) near the Fermi level. This provides direct electronic-structure evidence for the formation of effective chemisorption and interaction between CO_2_ and the catalyst surface.

However, key differences emerge in their PDOS features. Compared to Ni_5_/DVG, the Ni_4_Cu/DVG system exhibits a distinct modification in the electronic state distribution near the Fermi level. The introduction of Cu not only contributes additional electronic states but, more importantly, modulates the d-band electronic structure of the neighboring Ni atoms. This modulation directly influences the ability of the active sites to provide electronic back-donation into the anti-bonding orbitals of CO_2_, fundamentally determining the adsorption configuration, the adsorption strength of CO_2_, and consequently the ease of C=O bond activation. This analysis provides direct evidence for the electronic origin underlying the potentially superior catalytic performance of Ni_4_Cu/DVG.

#### 3.2.2. Exploration of Reaction Pathways

**Figure 4:**
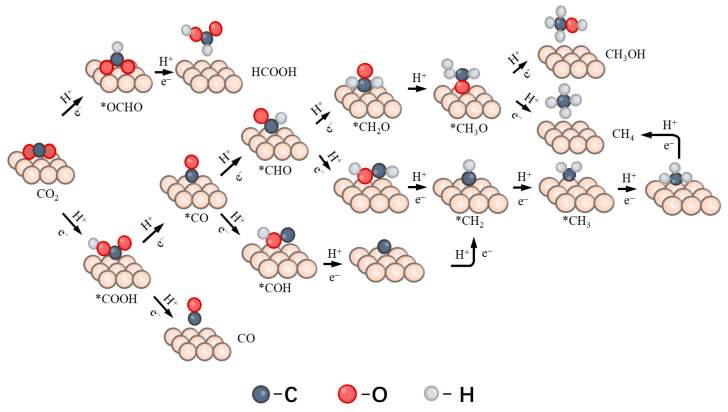
Schematic illustration of the reaction pathways for CO_2_ reduction reaction (CO_2_RR) to C_1_ products (CO, HCOOH, CH_3_OH, and CH_4_). The asterisk (*) denotes adsorbed species on the catalyst surface.

The electrocatalytic CO_2_ reduction reaction (CO_2_RR) involves multi-step proton-coupled electron transfer (PCET) processes, leading to a variety of possible products including CO, HCOOH, and CH_4_ (Figure 4).

**Figure 5:**
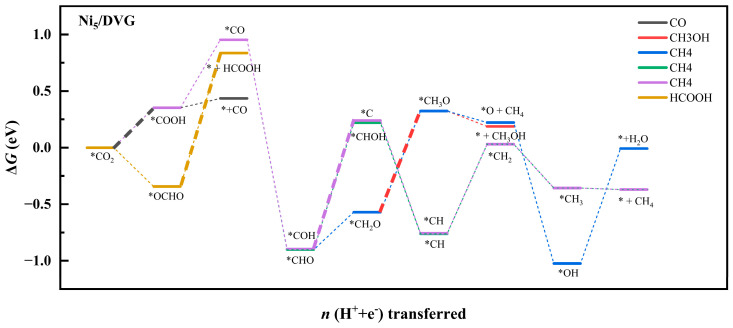
Reaction pathway diagram for CO_2_ reduction on the Ni_5_/dvg catalyst. The rate-determining steps (RDS) for each product are highlighted in bold, with corresponding energy barriers labeled in eV. Asterisk (*) denotes adsorbed intermediates.

**Figure 6:**
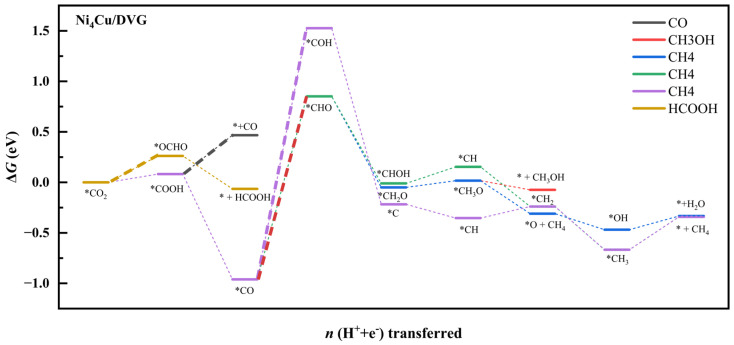
Reaction pathway diagram for CO_2_ reduction on the Ni_4_Cu/DVG catalyst. The RDS for each product are highlighted in bold, with corresponding energy barriers labeled in eV. Asterisk (*) denotes adsorbed intermediates.

Building upon our understanding of catalyst structural stability and the electronic structure of adsorbed CO_2_, we systematically investigated the performance and reaction pathways of CO_2_ reduction catalyzed by Ni_5_/DVG and Ni_4_Cu/DVG. By calculating the Gibbs free energies of key intermediates, we constructed free energy diagrams for all plausible reaction pathways (Figure 5 and Figure 6), thereby elucidating the differences in their catalytic activity and selectivity.

Comparison of the free energy diagrams for the reaction pathways on Ni_5_/DVG and Ni_4_Cu/DVG reveals that the energy barrier of the rate-determining step for HCOOH formation decreases significantly from 1.18 eV to 0.26 eV—a reduction of 0.92 eV. This substantial lowering of the energy barrier indicates a marked enhancement in reaction kinetics. The observed improvement in catalytic performance can be attributed to the modulation of the electronic structure induced by Cu alloying, which shares the same electronic origin as the enhanced stability of the Ni_4_Cu cluster on the support discussed earlier.

#### 3.2.3. Hydrogen Evolution Reaction (HER)

**Figure 7:**
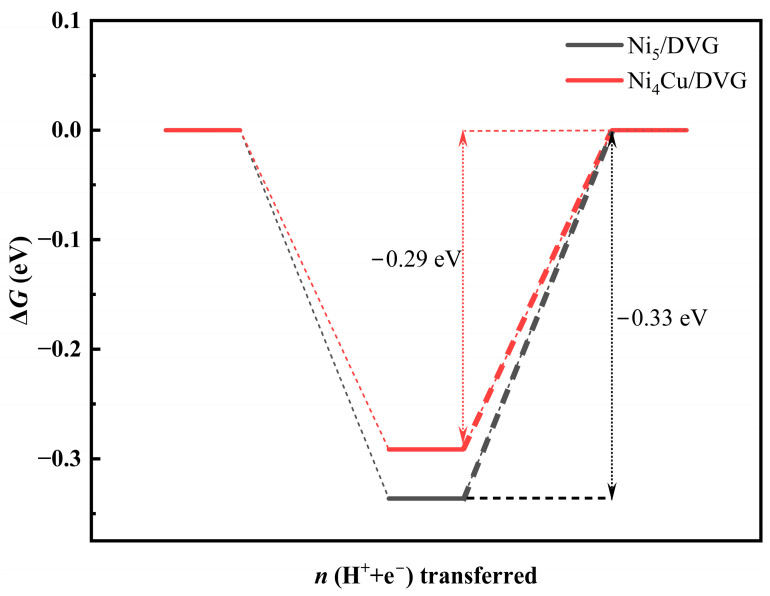
RDS energy barriers (eV) for HER.

In CO_2_RR, the hydrogen evolution reaction (HER) serves as the primary competing side reaction, directly determining product selectivity. To evaluate the kinetic competition, we calculated the HER (see Figure 7) and compared it with the RDS energy barriers for C_1_ products on two types of catalysts. The results indicate that on Ni_5_/DVG, the RDS energy barrier for HER (0.33 eV) is lower than those for C_1_ products (0.35 eV for CO and 1.18 eV for HCOOH), suggesting that H_2_ is the kinetically favorable product. On Ni_4_Cu/DVG, however, the energy barrier for HCOOH formation (0.26 eV) is lower than that for H_2_ desorption (0.29 eV), indicating that this catalyst exhibits a kinetic preference for the formate pathway, thereby effectively suppressing HER and enhancing selectivity for HCOOH.

### 3.3. Mechanism Analysis

**Figure 8:**
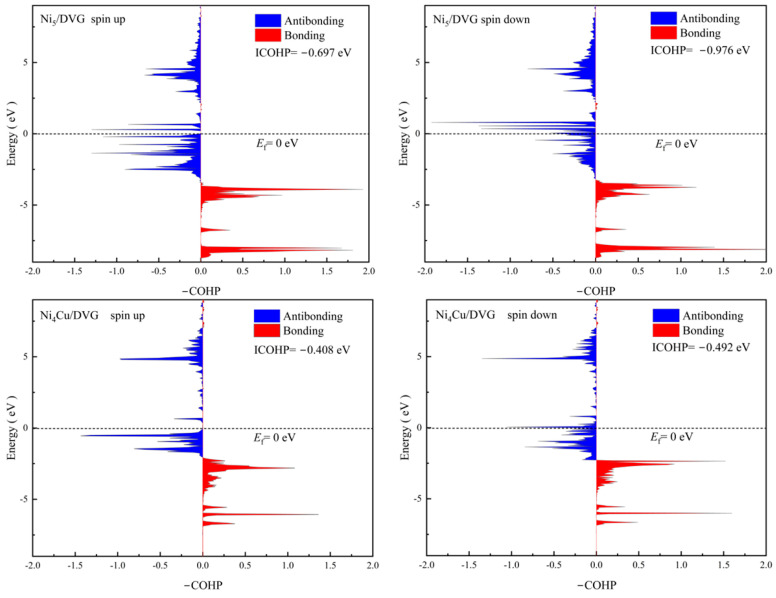
Crystal Orbital Hamilton Population (COHP) analysis for the Ni–O and Cu–O bonds upon *OCHO adsorption on Ni_5_/DVG and Ni_4_Cu/DVG.

To elucidate the regulatory mechanism governing the reaction pathway at the chemical bond level, we performed Crystal Orbital Hamilton Population (COHP) analysis on the adsorption behavior of the key intermediate *OCHO (Figure 8). This analysis directly reveals the bonding nature underlying the optimized catalytic performance of Ni_4_Cu/DVG. Integration of the COHP curve up to the Fermi level yields the integrated COHP (ICOHP), which serves as a quantitative measure of the overall bond strength. A larger absolute ICOHP value indicates stronger bonding between the atomic pairs.

For the critical metal-oxygen (M-O) bonds that determine the adsorption stability of the *OCHO intermediate, the integrated ICOHP values for the Ni-O bonds in the Ni_5_/DVG system are −0.697 eV and −0.976 eV, respectively. In contrast, the corresponding ICOHP values for the Cu-O bonds in the Ni_4_Cu/DVG system are −0.408 eV and −0.492 eV. A larger absolute ICOHP value corresponds to stronger net bonding. This comparison reveals that Ni_5_/DVG excessively stabilizes the *OCHO intermediate on the surface through stronger Ni-O bonding, which is unfavorable for subsequent transformation.

From an orbital distribution perspective, the bonding orbitals of Ni_5_/DVG are more fully occupied below the Fermi level, with relatively minor contributions from antibonding orbitals, further confirming its stronger bonding stability. Upon Cu doping, the bonding interaction of the Cu-O bond weakens, which appropriately reduces the adsorption strength of the *OCHO intermediate, thereby facilitating its desorption and subsequent transformation. This modulation effectively tailors the reaction pathway.

In summary, Ni_5_/DVG exhibits excessively strong adsorption bonding toward the *OCHO intermediate, whereas Ni_4_Cu/DVG achieves an optimized adsorption–desorption balance through modulation of the M-O bond strength. These findings provide a clear bonding-level understanding of the *OCHO transformation behavior in the electrocatalytic reduction of CO_2_.

## 4. Conclusions

Based on first-principles calculations, this work systematically investigated the electrocatalytic CO_2_ reduction performance of monometallic Ni_5_ and bimetallic Ni_4_Cu clusters anchored on DVG. The Ni_4_Cu/DVG catalyst exhibits higher thermodynamic stability, with a binding energy of −7.5 eV compared to −6.2 eV for Ni_5_/DVG, attributed to increased charge transfer and strengthening of Ni(Cu)–C interfacial bonding. Ni_4_Cu/DVG reduces the energy barrier of the rate-determining step for formic acid formation from 1.18 eV to 0.26 eV, indicating faster reaction kinetics. Mechanistic analysis reveals that Cu incorporation modulates the electronic structure of the active sites and weakens the M–O bond strength of the *OCHO intermediate. The integrated COHP (ICOHP) values are −0.408 eV/−0.492 eV for Cu–O bonds, compared to −0.697 eV/−0.976 eV for Ni–O bonds. This weakening suppresses excessive stabilization of the intermediate and facilitates subsequent conversion.

The calculated binding energy (−7.5 eV) and charge transfer analysis indicate strong interfacial interaction between the Ni_4_Cu cluster and the DVG support, suggesting the potential synthesizability of the catalyst. Experimental validation of its CO_2_RR performance remains an important aspect for future investigation. Notably, this study examined the Ni:Cu atomic ratio of 4:1; other compositions, such as Ni_3_Cu_2_ and Ni_2_Cu_3_, remain unexplored. The Ni/Cu ratio is important in modulating the electronic structure of the active sites and the adsorption behavior of key intermediates, including *OCHO and *H. Further optimization of this ratio may further clarify the structure–activity relationship and enhance catalytic performance. In addition, this alloying strategy may be extended to other transition metal combinations, providing a basis for the design of non-precious metal electrocatalysts for CO_2_ reduction.

## Figures and Tables

**Table 1:** Bader charge analysis of the catalysts. Positive values indicate electron loss (oxidation), while negative values indicate electron gain (reduction).

	Ni_5_	DVG
Ni_5_/DVG	1.326	−1.326
Ni_4_Cu/DVG	1.338	−1.338

## Data Availability

The raw data required to reproduce these findings cannot be shared at this time as the data also form part of an ongoing study.
